# Associations of serum lactate and lactate clearance with delirium in the early stage of ICU: a retrospective cohort study of the MIMIC-IV database

**DOI:** 10.3389/fneur.2024.1371827

**Published:** 2024-07-01

**Authors:** Xiangfeng Qian, Yi Sheng, Yinsheng Jiang, Yong’an Xu

**Affiliations:** ^1^Department of Emergency Medicine, Linping Campus, The Second Affiliated Hospital of Zhejiang University School of Medicine, Hangzhou, Zhejiang, China; ^2^Department of Emergency Medicine, The Second Affiliated Hospital Zhejiang University School of Medicine, Hangzhou, Zhejiang, China

**Keywords:** lactate, lactate clearance rate, delirium, MIMIC-IV, early stage of ICU

## Abstract

**Aim:**

This study aimed to investigate the associations of serum lactate level [within and after 24 h of the intensive care unit (ICU) admission] and lactate clearance rate with delirium and assess associations of lactate and lactate clearance rate with 30-day mortality in delirium patients.

**Methods:**

Data in this retrospective cohort study were extracted from the Medical Information Mart for Intensive Care IV (MIMIC-IV) database in 2012–2019. The associations of lactate and lactate clearance rate with delirium were explored through univariable and multivariable logistic regression analyses, whereas the associations of lactate and lactate clearance rate with 30-day mortality in delirium patients were investigated using univariable and multivariable Cox regression analyses. Subgroup analysis was performed for age, gender, sepsis, hypertension, sedative drug, ventilation, antibiotic drug, vasopressors, and the Sequential Organ Failure Assessment (SOFA) score. The evaluation indexes were odds ratios (ORs), hazard ratios (HRs), and 95% confidence intervals (CIs).

**Results:**

Among 7,812 (14.58%) eligible participants, 4,338 (8.19%) had delirium and 1,903 (24.36%) died within 30 days. After adjusting for covariates, patients with lactic acidosis (lactate level > 5 mmol/L and PH < 7.35) at T0 (within 24 h of the ICU admission) had higher odds of delirium (OR = 1.235, 95%CI: 1.105–1.382). Hyperlactatemia (lactate level 2–5 mmol/L and PH > 7.35) at T1 (after 24 h of the ICU admission) was also associated with higher odds of delirium (OR = 1.277, 95%CI: 1.126–1.447). Lactate clearance rate > 50% was linked to lower odds of delirium (OR = 0.705, 95%CI: 0.613–0.811), and this relationship was also observed in ≥65 years old, female, male, non-sepsis, sepsis, non-hypertension, non-sedative drug use, sedative drug use, ventilation, antibiotic drug use, use of vasopressors, and different SOFA score subgroups (all *p* < 0.05). Additionally, hyperlactatemia and lactic acidosis (whether at T0 or T1) may be potential risk factors for 30-day mortality in delirium patients, whereas lactate clearance rate ≥ 0 had a potential protective effect on 30-day mortality (all *p* < 0.05).

**Conclusion:**

Higher serum lactate levels in the early stage of the ICU were associated with a higher risk of delirium and subsequent mortality. Measures taken to increase the lactate clearance rate are necessary to reduce potential delirium or mortality risk in clinical settings. However, more evidence from prospective studies is needed to verify these findings.

## Introduction

Delirium is an acute psychiatric syndrome with the characteristics of a disturbance in attention and cognition (1). In the intensive care unit (ICU), delirium is a common complication, and its prevalence rates amount to approximately 22.0%, which has a strong influence on patients’ treatment during the ICU stay as well as the prognosis after discharge (2, 3). As the pathogenesis of delirium is still unclear, it is of great significance to explore the biological indexes for delirium identification in the early stage that could improve delirium prognosis and reduce disease burden.

It has been speculated that delirium may be related to the rupture of the blood–brain barrier (BBB) and that dynamically increased permeability is associated with neuroinflammation and lactate response (4). The serum lactate level on the ICU admission is commonly used as a biomarker to assess disease severity and predict outcomes in patients, such as sepsis, septic shock, general trauma, or traumatic brain injury (TBI) (5, 6). The elevated serum lactate levels are most likely correlated with a persistent oxygen delivery deficit or a damaged microcirculation resulting in tissue ischemia (7). In addition, lactate is an important metabolism substrate, and the abnormal change in its concentration can indicate an imbalance of cerebral metabolism, which can be used to predict neurological function impairment and outcomes (8). Research studies have reported that serum lactate metabolism was linked to several central nervous system (CNS) diseases (e.g., mild cognitive impairment and cognitive recovery after TBI) (9–11). A previous retrospective cohort study suggested that serum lactate level 1 h after surgery may be a predictor of postoperative delirium (POD) development in elderly trauma patients (12).

In recent years, the lactate clearance rate has been proposed to be a better biomarker for prognosis in critical patients compared to the time point lactate level. The lactate clearance rate is closely associated with capillary perfusion independent of hemodynamic variables, which can reflect treatment efficacy and disease progression over a period of time (13, 14). A retrospective study in patients with acute gastrointestinal (GI) bleeding validated that lactate clearance rate at 3-h intervals was useful for early prediction of mortality and other prognoses (15). A latest study based on the Medical Information Mart for Intensive Care (MIMIC) database showed high lactate clearance rate between 0 and 12 h of mechanical ventilation was associated with a decreased risk of 30-day mortality in ICU patients (7). Nevertheless, the associations of lactate clearance rate with delirium risk are unclear.

Herein, this study aimed to investigate the associations of lactate and lactate clearance rate with delirium in ICU patients and assess the associations of lactate and lactate clearance rate with short-term mortality in delirium patients, to provide some references for further exploration on early biomarkers of delirium and prognosis improvement.

## Methods

### Study design and population

This was a retrospective cohort study. Data of participants were extracted from the MIMIC-IV database. The MIMIC-IV is intended to support a wide variety of research in healthcare because it contains true hospitalized patients admitted to a tertiary academic medical center in Boston, MA, United States, in 2012–2019. Comprehensive data on laboratory measurements, medications administered, and vital signs of each patient when they stayed in the hospital were documented and included in the MIMIC-IV (16).

First, we included 53,569 patients hospitalized in the ICU for the first time from the database. The inclusion criteria were as follows: (1) aged ≥18 years old, (2) stayed in the ICU >1 day, (3) having information on serum lactate concentration examined between 0 and 24 h of the ICU admission (T0) and that after 24 h of the ICU admission (T1), and (4) not diagnosed with coma or delirium within 24-h stay. Patients were excluded if they had one of the following situations: dementia, psychoses, TBI, dyslexia, intellectual disability, nervous system diseases, or alcohol/drug abuse. Finally, 7,812 patients were eligible. The MIMIC-IV has been approved by Institutional Review Boards (IRBs) of the Massachusetts Institute of Technology (MIT) and the Beth Israel Deaconess Medical Center (BIDMC) (17). As this database is publicly available, the IRB of the hospital waived the ethical approval.

### Measurements of serum lactate and lactate clearance rate

We extracted information on serum lactate concentration (taken from arterial blood gases) at two time points, including the first examination between 0 and 24 h of the ICU admission (T0 lactate) and the first examination after 24 h of admission (T1 lactate). Then, the serum lactate levels were divided into three categories according to previous studies (18, 19): normal lactate level (<2 mmol/L), hyperlactatemia (2–5 mmol/L and PH > 7.35), and lactic acidosis (>5 mmol/L and PH < 7.35). In addition, the lactate clearance rate was calculated through the formula: lactate clearance rate = (T0 lactate − T1 lactate)/T0 lactate × 100%, and it was categorized into <0, 0–50, and > 50% levels (20).

### Diagnosis of delirium

Two steps were conducted to identify delirium. First, the Richmond Agitation and Sedation Scale (RASS) was used to assess delirium. An individual with an RASS score < −3 was recognized as being in a coma and did not conform to the standard of the next step assessment (21). Then, delirium in eligible persons (with RASS score ≥ −3) was assessed using the Confusion Assessment Method for the ICU (CAM-ICU). The CAM-ICU consists of four features, including feature 1: acute change or fluctuating course of mental status; feature 2: inattention; feature 3: disorganized thinking; and feature 4: altered level of consciousness (LOC) (22). When features 1 and 2 are present with either feature 3 or 4, patients were considered CAM-ICU positive and have a delirious status (23).

### Outcomes and follow-up period

The study outcomes were as follows: (1) occurrence of delirium after 24 h of the ICU admission in the total population and (2) 30-day mortality in patients with delirium. The site of both delirium and mortality events included during ICU stay or after discharge. In-hospital information was recorded by the hospital department, and out-of-hospital information was recorded by the Social Security Bureau. Hence, the information on 30-day mortality in the MIMIC-IV was extracted from the patients’ self-case. The follow-up started from the first time of ICU admission and ended when patients were discharged or died or 30 days after the ICU admission. Patients with outcome events after 30 days were not censored.

### Variable extraction

We also extracted the following variables from the MIMIC-IV database: age, gender, insurance, heart rate (HR), diastolic blood pressure (DBP), systolic blood pressure (SBP), respiratory rate (RR), temperature, SPO_2_, pH, white blood cell (WBC), red cell distribution width (RDW), platelet, hematocrit, creatinine (Cr), international normalized ratio (INR), prothrombin time (PT), blood urea nitrogen (BUN), bicarbonate, sodium (Na), potassium (K), chloride, glucose, the Sequential Organ Failure Assessment (SOFA), the Charlson Comorbidity Index (CCI), the Glasgow Coma Scale (GCS), sepsis, cardiovascular disease (CVD), diabetes mellitus (DM), hypertension, chronic kidney disease (CKD), liver disease, depression, ventilator-associated pneumonia (VAP), ventilation, use of vasopressors, sedative drug use, and antibiotic drug use. Additionally, information on these variables was measured and recorded for the first time within 24 h of the ICU admission.

### Statistical analysis

The continuous variables are presented as median and quartiles [M (Q1, Q3)]. The Wilcoxon rank-sum test was used to compare the difference between the delirium and non-delirium groups. Frequency and composition ratio [*N* (%)] was used to describe classified variables, and the chi-square test (χ^2^) was used for comparison between the delirium and non-delirium groups.

A univariable logistic regression analysis was used to screen covariates, and variables significantly associated with delirium were recognized as potential covariates and included in the adjustment of multivariate models. An exploration of the associations of serum lactate and lactate clearance rate with delirium was made through univariable and multivariable logistic regression analyses. The evaluation indexes were odds ratios (ORs) and 95% confidence intervals (CIs). The multicollinearity among study variables in logistic regression models was assessed through the variance inflation factor (VIF) method (Supplementary Table S1). The average VIF value less than 5 indicates no multicollinearity.

The relationships of lactate and lactate clearance rate with 30-day mortality in patients with delirium were found using univariable and multivariable Cox regression analyses. Similarly, univariable Cox regression analysis was used for the screening of covariates. The Cox model is a classical technique for performing analyses on time-to-event data (24). The evaluation indexes were hazard ratios (HRs) and 95% CIs, namely, the estimated risk of a given endpoint associated with a specific risk factor. The Cox proportional hazard model fit statistics were assessed by the Schoenfeld individual test (Supplementary Figure S1).

The Kaplan–Meier (KM) curves were drawn to evaluate mortality distributions among patients with different serum lactate levels. Among the multivariate models, model 1 adjusted for demographic and vital signs variables (including race, HR, DBP, SBP, RR, SPO_2_, and pH); model 2 adjusted for demographic, vital signs, and laboratory examination variables (including race, HR, DBP, SBP, RR, SPO_2_, pH, RDW, platelet, Cr, INR, PT, BUN, bicarbonate, and Na); model 3 adjusted for all selected covariates (including race, HR, DBP, SBP, RR, SPO_2_, pH, RDW, platelet, Cr, INR, PT, BUN, bicarbonate, Na, SOFA, CCI, GCS, sepsis, CVD, liver disease, ventilation, use of vasopressors, VAP, sedative drug use, and antibiotic drug use). In addition, subgroup analyses of age, gender, sepsis, hypertension, sedative drug use, ventilation, antibiotic drug use, use of vasopressors, and the SOFA score were performed. Two-sided *p* < 0.05 indicates a significant difference. Statistics analyses were completed using SAS 9.4 (SAS Institute., Cary, NC, United States) and R version 4.2.3 (2023-04-17 ucrt).

## Results

### Characteristics of eligible participants

**Figure 1:**
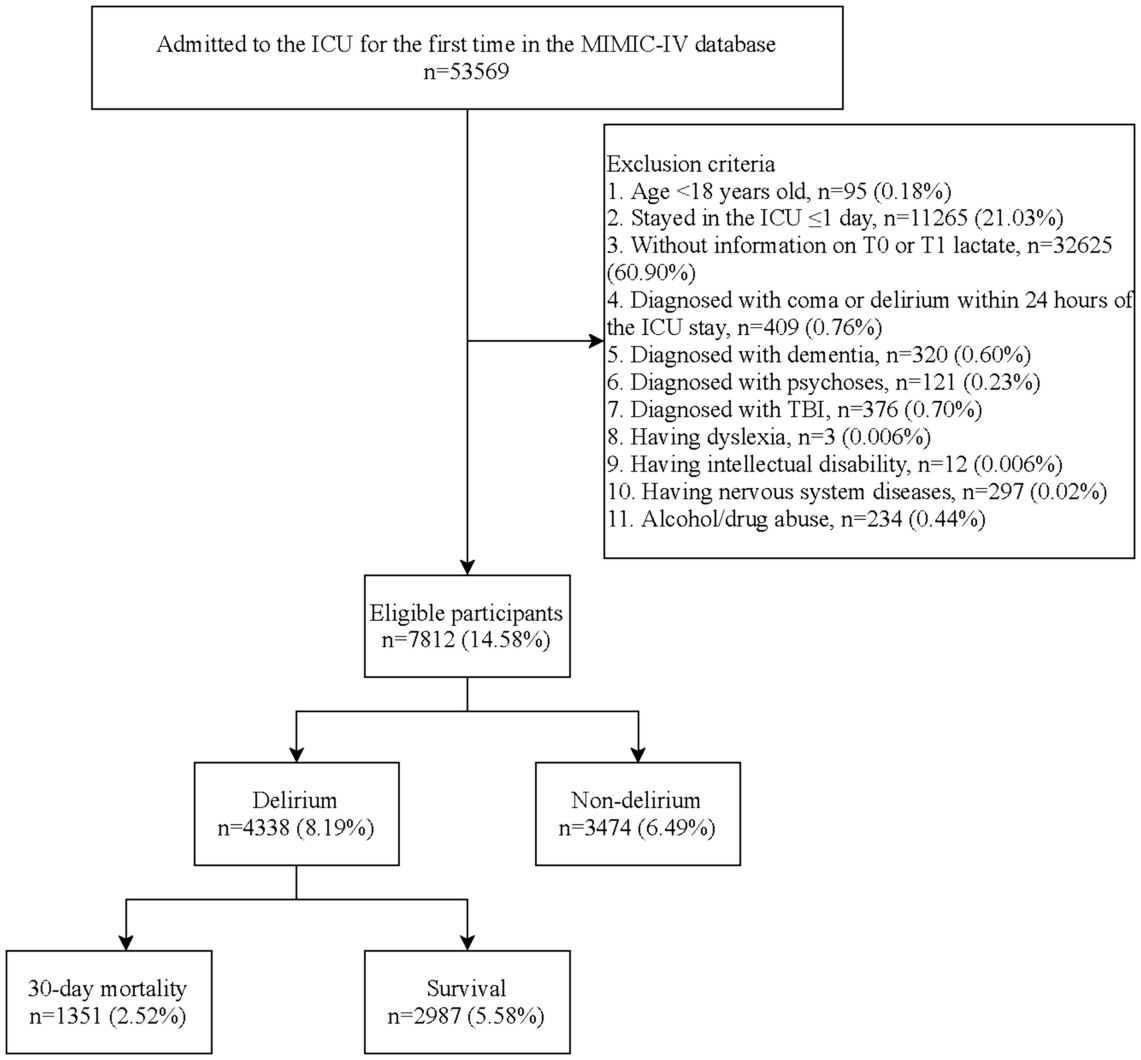
Flowchart of the study participants screening.

The flowchart of the study process is shown in Figure 1. A total of 53,569 individuals were admitted to the ICU for the first time in the MIMIC-IV database. Patients aged <18 years old (*n* = 95), stayed in the ICU ≤1 day (*n* = 11,265), without information on T0 or T1 lactate (*n* = 32,625), diagnosed with coma or delirium within 24 h of the ICU stay (*n* = 409), diagnosed with dementia (*n* = 320), psychoses (*n* = 121), TBI (*n* = 376), dyslexia (*n* = 3), intellectual disability (*n* = 12), nervous system diseases (*n* = 297), or alcohol/drug abuse (*n* = 234) were excluded. Finally, 7,812 patients were eligible.

Table 1 shows the characteristics of patients between the delirium and the non-delirium groups. The median age of the total study population was 65 years old, and 3,327 (42.59%) were women. Between the non-delirium and delirium groups, the median values of T0 lactate (2.00 mmol/L vs. 2.10 mmol/L), T1 lactate (1.40 mmol/L vs. 1.50 mmol/L), and lactate clearance rate (26.92% vs. 22.22%) were, respectively, different. In addition, race, HR, DBP, SBP, RR, SPO_2_, pH, RDW, platelet, Cr, INR, PT, BUN, bicarbonate, Na, SOFA, CCI, GCS, sepsis, CVD, liver disease, ventilation, use of vasopressors, VAP, sedative drug use, and antibiotic drug use were significantly different between these two groups (*p* < 0.05).

**Table 1:** Characteristics of eligible participants.

Variables	Total (*n* = 7,812)	Non-delirium (*n* = 3,474)	Delirium (*n* = 4,338)	Statistic	*p*
Age, years, M (Q_1_, Q_3_)	65.00 (54.00, 76.00)	65.00 (54.00, 75.00)	65.00 (54.00, 76.00)	*Z* = −0.196	0.845
Gender, *n* (%)				χ^2^ = 0.578	0.447
Female	3,327 (42.59)	1,463 (42.11)	1,864 (42.97)		
Male	4,485 (57.41)	2,011 (57.89)	2,474 (57.03)		
Race, *n* (%)				χ^2^ = 27.178	<0.001
White	5,022 (64.29)	2,343 (67.44)	2,679 (61.76)		
Other	2,790 (35.71)	1,131 (32.56)	1,659 (38.24)		
Insurance, *n* (%)				χ^2^ = 3.896	0.143
Medicaid	550 (7.04)	223 (6.42)	327 (7.54)		
Medicare	3,486 (44.62)	1,551 (44.65)	1,935 (44.61)		
Other	3,776 (48.34)	1,700 (48.93)	2,076 (47.86)		
HR, bpm, M (Q_1_, Q_3_)	90.00 (78.00, 107.00)	89.00 (78.00, 104.00)	92.00 (79.00, 108.00)	*Z* = −4.143	<0.001
DBP, mmHg, M (Q_1_, Q_3_)	64.00 (54.00, 76.00)	63.50 (54.00, 75.00)	65.00 (54.00, 78.00)	*Z* = −3.202	0.001
SBP, mmHg, M (Q_1_, Q_3_)	117.00 (102.00, 135.00)	116.00 (101.00, 133.00)	118.00 (103.00, 136.00)	*Z* = −3.203	0.001
RR, insp/min, M (Q_1_, Q_3_)	19.00 (16.00, 24.00)	18.00 (15.00, 23.00)	20.00 (16.00, 24.00)	*Z* = −7.589	<0.001
Temperature, °C, M (Q_1_, Q_3_)	36.70 (36.33, 37.11)	36.67 (36.33, 37.06)	36.72 (36.39, 37.17)	*Z* = −4.422	<0.001
SPO_2_, %, M (Q_1_, Q_3_)	98.00 (95.00, 100.00)	98.00 (95.00, 100.00)	98.00 (95.00, 100.00)	*Z* = −3.126	0.002
pH, M (Q_1_, Q_3_)	7.36 (7.28, 7.42)	7.37 (7.30, 7.42)	7.34 (7.26, 7.41)	*Z* = −10.707	<0.001
WBC, K/UL, M (Q_1_, Q_3_)	12.40 (8.60, 17.60)	12.10 (8.50, 17.20)	12.70 (8.70, 17.90)	*Z* = −2.938	0.003
RDW, %, M (Q_1_, Q_3_)	14.70 (13.60, 16.40)	14.55 (13.50, 16.00)	14.80 (13.70, 16.60)	*Z* = −6.908	<0.001
Platelet, K/UL, M (Q_1_, Q_3_)	175.00 (121.00, 247.00)	178.00 (126.00, 252.00)	173.00 (116.00, 243.00)	*Z* = −3.334	<0.001
Hematocrit, %, M (Q_1_, Q_3_)	32.00 (27.00, 37.10)	32.00 (27.10, 36.90)	32.00 (27.00, 37.27)	*Z* = −0.367	0.714
Cr, mg/dL, M (Q_1_, Q_3_)	1.10 (0.80, 1.80)	1.00 (0.80, 1.60)	1.20 (0.80, 1.90)	*Z* = −8.798	<0.001
INR, M (Q_1_, Q_3_)	1.40 (1.20, 1.70)	1.30 (1.20, 1.60)	1.40 (1.20, 1.70)	*Z* = −2.566	0.010
PT, s, M (Q_1_, Q_3_)	14.90 (13.00, 18.30)	14.80 (13.00, 17.80)	15.00 (13.00, 18.70)	*Z* = −1.871	0.061
BUN, mg/dL, M (Q_1_, Q_3_)	22.00 (15.00, 37.00)	21.00 (14.00, 33.00)	24.00 (16.00, 40.75)	*Z* = −8.813	<0.001
Bicarbonate, mEq/L, M (Q_1_, Q_3_)	21.00 (18.00, 24.00)	22.00 (19.00, 24.00)	21.00 (18.00, 24.00)	*Z* = −6.842	<0.001
Na, mEq/L, M (Q_1_, Q_3_)	138.00 (134.00, 141.00)	137.00 (134.00, 140.00)	138.00 (135.00, 141.00)	*Z* = −7.087	<0.001
K, mEq/L, M (Q_1_, Q_3_)	4.20 (3.80, 4.80)	4.30 (3.80, 4.80)	4.20 (3.70, 4.80)	*Z* = −1.799	0.072
Chloride, mEq/L, M (Q_1_, Q_3_)	104.00 (100.00, 108.00)	104.00 (100.00, 108.00)	104.00 (100.00, 108.00)	*Z* = −0.607	0.544
Glucose, mg/dL, M (Q_1_, Q_3_)	140.00 (112.00, 183.00)	138.00 (111.00, 179.00)	141.00 (112.00, 185.00)	*Z* = −1.881	0.060
SOFA, M (Q_1_, Q_3_)	3.00 (1.00, 5.00)	3.00 (1.00, 5.00)	3.00 (1.00, 6.00)	*Z* = −10.285	<0.001
CCI, M (Q_1_, Q_3_)	3.00 (1.00, 5.00)	3.00 (1.00, 4.00)	3.00 (1.00, 5.00)	*Z* = −6.035	<0.001
GCS, M (Q_1_, Q_3_)	15.00 (15.00, 15.00)	15.00 (15.00, 15.00)	15.00 (15.00, 15.00)	*Z* = −5.700	<0.001
Sepsis, *n* (%)				χ^2^ = 185.333	<0.001
No	4,517 (57.82)	2,304 (66.32)	2,213 (51.01)		
Yes	3,295 (42.18)	1,170 (33.68)	2,125 (48.99)		
CVD, *n* (%)				χ^2^ = 9.112	0.003
No	3,223 (41.26)	1,368 (39.38)	1,855 (42.76)		
Yes	4,589 (58.74)	2,106 (60.62)	2,483 (57.24)		
DM, *n* (%)				χ^2^ = 1.413	0.235
No	5,348 (68.46)	2,354 (67.76)	2,994 (69.02)		
Yes	2,464 (31.54)	1,120 (32.24)	1,344 (30.98)		
Hypertension, *n* (%)				χ^2^ = 2.218	0.136
No	4,720 (60.42)	2,067 (59.50)	2,653 (61.16)		
Yes	3,092 (39.58)	1,407 (40.50)	1,685 (38.84)		
CKD, *n* (%)				χ^2^ = 1.496	0.221
No	6,355 (81.35)	2,847 (81.95)	3,508 (80.87)		
Yes	1,457 (18.65)	627 (18.05)	830 (19.13)		
Liver disease, n (%)				χ^2^ = 38.862	<0.001
No	6,267 (80.22)	2,896 (83.36)	3,371 (77.71)		
Yes	1,545 (19.78)	578 (16.64)	967 (22.29)		
Depression, *n* (%)				χ^2^ = 6.228	0.013
No	7,227 (92.51)	3,185 (91.68)	4,042 (93.18)		
Yes	585 (7.49)	289 (8.32)	296 (6.82)		
Ventilation, *n* (%)				χ^2^ = 483.178	<0.001
Mechanical ventilation	4,850 (62.08)	1,716 (49.40)	3,134 (72.25)		
Supplemental oxygen	2,644 (33.85)	1,507 (43.38)	1,137 (26.21)		
None	318 (4.07)	251 (7.23)	67 (1.54)		
Vasopressors, *n* (%)				χ^2^ = 241.645	<0.001
No	2,300 (29.44)	1,334 (38.40)	966 (22.27)		
Yes	5,512 (70.56)	2,140 (61.60)	3,372 (77.73)		
VAP, *n* (%)				χ^2^ = 329.321	<0.001
No	7,148 (91.5)	3,401 (97.90)	3,747 (86.38)		
Yes	664 (8.5)	73 (2.10)	591 (13.62)		
Sedative drug, *n* (%)				χ^2^ = 729.083	<0.001
No	1,625 (20.8)	1,204 (34.66)	421 (9.70)		
Yes	6,187 (79.2)	2,270 (65.34)	3,917 (90.30)		
Antibiotic drug, *n* (%)				χ^2^ = 325.903	<0.001
No	798 (10.22)	595 (17.13)	203 (4.68)		
Yes	7,014 (89.78)	2,879 (82.87)	4,135 (95.32)		
T0 lactate, mmol/L, M (Q_1_, Q_3_)	2.10 (1.40, 3.30)	2.00 (1.30, 3.10)	2.10 (1.40, 3.50)	*Z* = −3.240	0.001
T1 lactate, mmol/L, M (Q_1_, Q_3_)	1.50 (1.10, 2.20)	1.40 (1.10, 2.00)	1.50 (1.10, 2.40)	*Z* = −7.877	<0.001
Lactate clearance rate, %, M (Q_1_, Q_3_)	25.00 (−8.33, 50.00)	26.92 (−6.25, 51.27)	22.22 (−9.15, 48.00)	*Z* = −3.878	<0.001
T0 lactate, *n* (%)				χ^2^ = 41.876	<0.001
Normal lactate level	3,649 (46.71)	1,627 (46.83)	2,022 (46.61)		
Hyperlactatemia	3,236 (41.42)	1,522 (43.81)	1,714 (39.51)		
Lactic acidosis	927 (11.87)	325 (9.36)	602 (13.88)		
T1 lactate, *n* (%)				χ^2^ = 67.316	<0.001
Normal lactate level	5,434 (69.56)	2,577 (74.18)	2,857 (65.86)		
Hyperlactatemia	1,898 (24.3)	736 (21.19)	1,162 (26.79)		
Lactic acidosis	480 (6.14)	161 (4.63)	319 (7.35)		
Lactate clearance rate, %, *n* (%)				χ^2^ = 10.683	0.005
<0	2,522 (32.28)	1,069 (30.77)	1,453 (33.49)		
0–50	3,449 (44.15)	1,533 (44.13)	1,916 (44.17)		
>50	1,841 (23.57)	872 (25.10)	969 (22.34)		
30-day mortality, *n* (%)				χ^2^ = 243.608	<0.001
No	5,909 (75.64)	2,922 (84.11)	2,987 (68.86)		
Yes	1,903 (24.36)	552 (15.89)	1,351 (31.14)		

Z: The Wilcoxon rank-sum test, χ^2^: chi-square test. M, Median; Q_1_, First quartile; Q_3_, Third quartile; HR, Heart rate; DBP, Diastolic blood pressure; SBP, Systolic blood pressure; RR, Respiratory rate; WBC, White blood cell; RDW, Red cell distribution width; Cr, Creatinine; INR, International normalized ratio; PT, Prothrombin time; BUN, Blood urea nitrogen; Na, Sodium; K, Potassium; SOFA, The Sequential organ failure assessment; CCI, The Charlson comorbidity index; GCS, The Glasgow Coma Scale; CVD, Cardiovascular disease; DM, Diabetes mellitus; CKD, Chronic kidney disease; VAP, Ventilator-associated pneumonia; T0, Serum lactate concentration examined at the ICU admission; T1, The first examination of serum lactate concentration within 24 h of the ICU admission.

### Associations of lactate and lactate clearance rate with delirium

**Figure 2:**
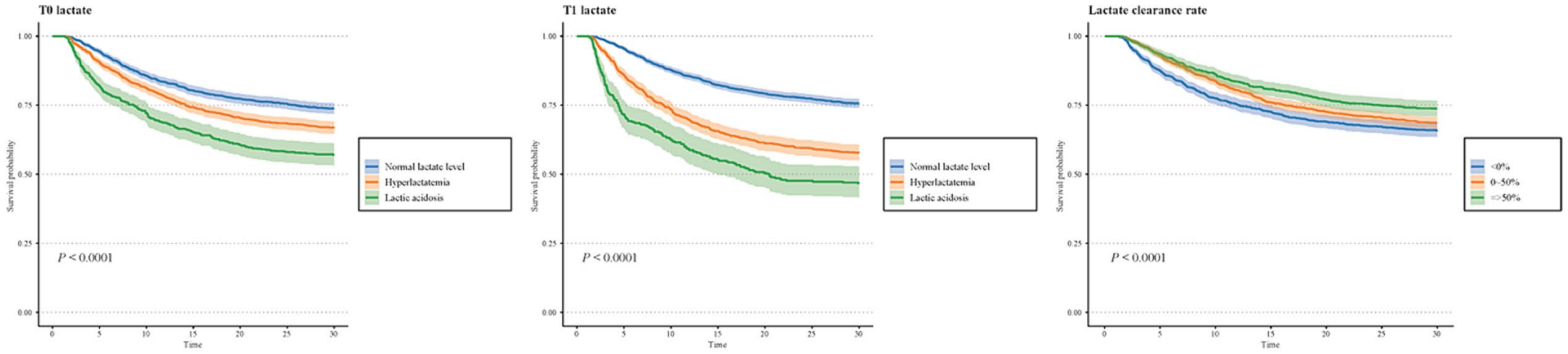
Associations of T0 lactate, T1 lactate, and lactate clearance rate with delirium in subgroups of age, gender, sepsis, hypertension, sedative drug, ventilation, antibiotic drug, vasopressors, and SOFA score.

We explored the associations of serum lactate levels and lactate clearance rate with delirium (Table 2). After adjusting for all covariates, the odds of delirium increased by 1.062 with T1 lactate elevated by 1 mmol/L (95%CI: 1.001–1.126), whereas the odds of delirium decreased by 0.081 with lactate clearance rate elevated by 1% (95%CI: 0.873–0.967). Patients with lactic acidosis at T0 seemed to have higher odds of delirium compared to those who had normal lactate levels (OR = 1.235, 95%CI: 1.105–1.382). Hyperlactatemia at T1 was also positively associated with delirium odds compared to normal lactate levels (OR = 1.277, 95%CI: 1.126–1.447). Lactate clearance rate > 50 may be a potential protective factor for delirium in ICU patients (OR = 0.705, 95%CI: 0.613–0.811). In addition, it was clearly shown in Figure 2 that patients with lactic acidosis (both at T0 and T1) had the lowest survival probability, followed by those with hyperlactatemia (all *p* < 0.05).

**Table 2:** Associations of lactate and lactate clearance rate with delirium.

Exposure	Model 1		Model 2		Model 3	
	OR (95% CI)	*p*	OR (95% CI)	*p*	OR (95% CI)	*p*
T0 lactate	1.070 (1.015–1.127)	0.011	1.047 (0.989–1.108)	0.112	0.965 (0.907–1.027)	0.266
T1 lactate	1.138 (1.079–1.200)	<0.001	1.102 (1.044–1.164)	<0.001	1.062 (1.001–1.126)	0.045
Lactate clearance rate	0.913 (0.870–0.958)	<0.001	0.916 (0.872–0.962)	<0.001	0.919 (0.873–0.967)	0.001
T0 lactate						
Normal lactate level	Ref		Ref		Ref	
Hyperlactatemia	1.645 (1.414–1.914)	<0.001	1.220 (1.039–1.432)	0.015	1.088 (0.909–1.302)	0.359
Lactic acidosis	1.104 (1.004–1.214)	0.042	1.161 (1.053–1.280)	0.003	1.235 (1.105–1.382)	<0.001
T1 lactate						
Normal lactate level	Ref		Ref		Ref	
Hyperlactatemia	1.424 (1.280–1.584)	<0.001	1.370 (1.229–1.528)	<0.001	1.277 (1.126–1.447)	<0.001
Lactic acidosis	1.787 (1.468–2.176)	<0.001	1.364 (1.114–1.671)	0.003	1.079 (0.859–1.354)	0.514
Lactate clearance rate						
<0	Ref		Ref		Ref	
0–50	0.920 (0.829–1.020)	0.113	0.883 (0.795–0.981)	0.021	0.926 (0.824–1.042)	0.203
>50	0.818 (0.724–0.923)	0.001	0.729 (0.644–0.826)	<0.001	0.705 (0.613–0.811)	<0.001

OR, Odds ratio; CI, Confidence interval; T0, Serum lactate concentration examined at the ICU admission; T1, The first examination of serum lactate concentration within 24 h of the ICU admission; Ref, Reference. Mode1 1: adjusted for race, HR, DBP, SBP, RR, SPO_2_, and pH; Model 2: adjusted for race, HR, DBP, SBP, RR, SPO_2_, pH, RDW, platelet, Cr, INR, PT, BUN, bicarbonate, and Na; Model 3: adjusted for race, HR, DBP, SBP, RR, SPO_2_, pH, RDW, platelet, Cr, INR, PT, BUN, bicarbonate, Na, SOFA, CCI, GCS, sepsis, CVD, liver disease, ventilation, use of vasopressors, VAP, sedative drug use, and antibiotic drug use.

Furthermore, the association of lactate clearance rate with delirium in patients who had different T0 lactate levels was assessed. Supplementary Figure S2 shows that among patients with hyperlactatemia/lactic acidosis at T0, the proportion of delirium individuals gradually decreased along with the increased lactate clearance rate. Similarly, after adjusting for covariates, elevated lactate clearance rate was significantly associated with decreased odds of delirium in patients with hyperlactatemia/lactic acidosis at T0 (OR = 0.901, 95%CI: 0.835–0.973). Compared to lactate clearance rate < 0, lactate clearance rates of 0–50% (OR = 0.792, 95%CI: 0.643–0.976) and > 50% (OR = 0.620, 95%CI: 0.500–0.768) were both linked to lower odds of delirium in patients with hyperlactatemia/lactic acidosis at T0.

### Associations of lactate and lactate clearance rate with delirium in subgroups

**Figure 3:**
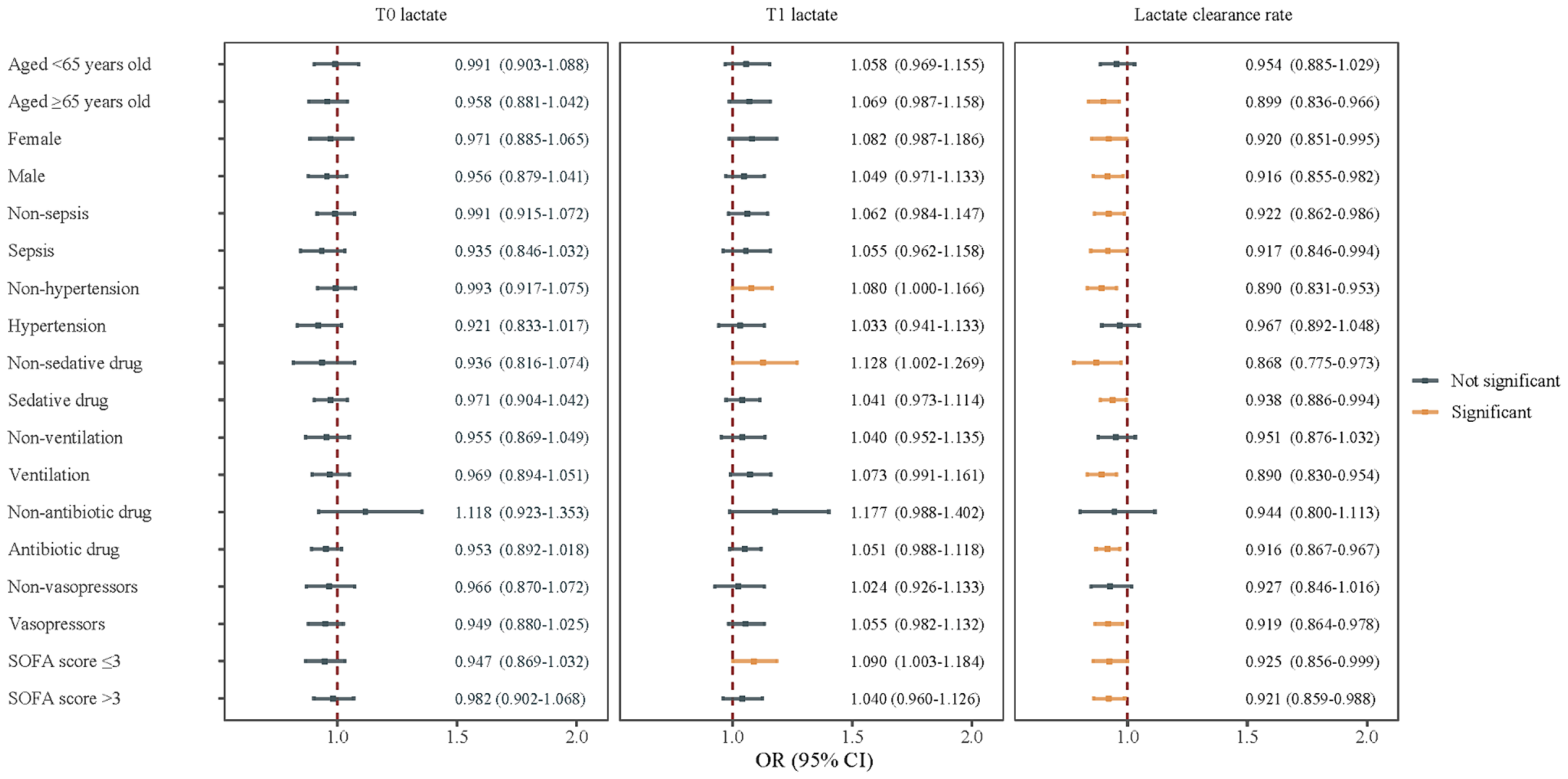
Associations of lactate and lactate clearance rate with delirium in subgroups of age, gender, sepsis, hypertension, sedative drug, ventilation, antibiotic drug, vasopressors, and SOFA score.

The relationships between lactate/lactate clearance rate and delirium were also explored in subgroups of age, gender, sepsis, hypertension, sedative drugs, ventilation, antibiotic drugs, vasopressors, and SOFA score (divided according to the median values) (Figure 3). T1 lactate had a positive association with delirium in patients without hypertension (OR = 1.080, 95%CI: 1.000–1.166), not use sedative drug (OR = 1.128 95%CI: 1.002–1.269), or with SOFA score ≤ 3 (OR = 1.090, 95%CI: 1.003–1.184). In addition, a higher lactate clearance rate was associated with lower odds of delirium in people aged ≥65 years old, female, male, non-sepsis, sepsis, non-hypertension, non-sedative drug use, sedative drug use, ventilation, antibiotic drug use, use of vasopressors, SOFA score > 3, and SOFA score ≤ 3 subgroups (all *p* < 0.05).

### Associations of lactate and lactate clearance rate with 30-day mortality in delirium patients

We further investigated the associations of lactate and lactate clearance rate with 30-day mortality in patients with delirium (Table 3). After adjusting for all covariates, T0 lactate (OR = 1.152, 95%CI: 1.096–1.211) and T1 lactate (OR = 1.423, 95%CI: 1.370–1.479) were both positively associated with 30-day mortality, whereas a higher lactate clearance rate was linked to a lower risk of 30-day mortality in delirium patients (OR = 0.855, 95%CI: 0.826–0.886). Compared to normal lactate levels, hyperlactatemia and lactic acidosis (whether at T0 or T1) were potential risk factors for 30-day mortality in delirium, while lactate clearance rate ≥ 0 had a potential protective value for 30-day mortality (all *p* < 0.05).

**Table 3:** Associations of lactate and lactate clearance rate with 30-day mortality in patients with delirium.

Variables	Model 1		Model 2		Model 3	
	HR (95% CI)	*p*	HR (95% CI)	*p*	HR (95% CI)	*p*
T0 lactate	1.157 (1.107–1.210)	<0.001	1.152 (1.096–1.211)	<0.001	1.132 (1.074–1.193)	<0.001
T1 lactate	1.433 (1.383–1.485)	<0.001	1.423 (1.370–1.479)	<0.001	1.409 (1.355–1.465)	<0.001
Lactate clearance rate	0.863 (0.834–0.892)	<0.001	0.855 (0.826–0.886)	<0.001	0.861 (0.831–0.891)	<0.001
T0 lactate						
Normal lactate level	Ref		Ref		Ref	
Hyperlactatemia	1.265 (1.123–1.425)	<0.001	1.251 (1.106–1.416)	<0.001	1.194 (1.054–1.353)	0.005
Lactic acidosis	1.586 (1.343–1.872)	<0.001	1.568 (1.315–1.869)	<0.001	1.448 (1.211–1.731)	<0.001
T1 lactate						
Normal lactate level	Ref		Ref		Ref	
Hyperlactatemia	1.898 (1.688–2.134)	<0.001	1.726 (1.529–1.949)	<0.001	1.657 (1.466–1.873)	<0.001
Lactic acidosis	2.615 (2.195–3.114)	<0.001	2.446 (2.042–2.929)	<0.001	2.275 (1.895–2.730)	<0.001
Lactate clearance rate						
<0	Ref		Ref		Ref	
0–50	0.869 (0.772–0.977)	0.019	0.883 (0.784–0.994)	0.040	0.861 (0.764–0.970)	0.014
>50	0.624 (0.534–0.728)	<0.001	0.665 (0.567–0.781)	<0.001	0.638 (0.543–0.750)	<0.001

HR: hazard ratio, CI: confidence interval, T0: serum lactate concentration examined at the ICU admission, T1: the first examination of serum lactate concentration within 24 h of the ICU admission, Ref: reference. Mode1 1: adjusted for race, HR, DBP, SBP, RR, SPO_2_, and pH; Model 2: adjusted for race, HR, DBP, SBP, RR, SPO_2_, pH, RDW, platelet, Cr, INR, PT, BUN, bicarbonate, and Na; and Model 3: adjusted for race, HR, DBP, SBP, RR, SPO_2_, pH, RDW, platelet, Cr, INR, PT, BUN, bicarbonate, Na, SOFA, CCI, GCS, sepsis, CVD, liver disease, ventilation, use of vasopressors, VAP, sedative drug use, and antibiotic drug use.

## Discussion

The current research investigated the associations of lactate and lactate clearance rate with delirium among ICU patients and the associations of lactate and lactate clearance rate with 30-day mortality in delirium patients. The results showed that compared to patients with normal lactate levels, those who had lactic acidosis at T0 or hyperlactatemia at T1 seemed to have higher odds of delirium. On the contrary, a lactate clearance rate > 50 was associated with lower odds of delirium. In addition, elevated T0 lactate and T1 lactate were both potential risk factors for 30-day mortality in patients with delirium, whereas a higher lactate clearance rate was linked to a lower risk of 30-day mortality.

To the best of our knowledge, it was the first time to investigate the association of serum lactate at different time points and lactate clearance rate with the occurrence of delirium in the general patients who stayed in the ICU. A previous retrospective cohort study on elderly trauma patients from a level 1 single trauma center showed that serum lactate levels on ICU admission and 1 h after surgery were predictors of postoperative delirium (12). Wang et al. (25) performed a prospective observational study and identified postoperative lactate levels as an independent predictor of delirium in patients who underwent cardiac surgery at the Nanjing Drum Tower Hospital, China. In the present study, we found serum lactate level after 24 h of ICU admission (T1 lactate level) was a potential risk factor for delirium. Compared to previous research studies, our findings relatively supplemented the association of serum lactate level with delirium among the ICU general population. In addition, the study participants were extracted from the MIMIC-IV database, which contains a large sample of true hospitalized patients in the United States. Unfortunately, according to our results, the odds of delirium were not significantly increased along with the elevated T0 lactate levels, indicating there may be no linear relationship between them. Similarly, Liu et al. (8) demonstrated that the serum lactate at ICU admission was not correlated with neurological function impairment in adult patients under general anesthesia elective neurosurgery after the surgery at The Seventh Medical Center of the General Hospital of the People’s Liberation Army of China. In the current research, 46.71% of participants had normal lactate levels at T0 (<2 mmol/L) and 41.42% had hyperlactatemia (2–5 mmol/L and pH > 7.35), whereas patients after the surgery in the study by Liu had higher serum lactate than normal commonly (3.4–4.1 mmol/L) (8). In fact, fluid transfusion and vasoactive agent use might affect serum lactate levels during the ICU stay; thus, we adjusted for sedative drug use and antibiotic drug use in multivariate models and found that compared to patients with normal T0 lactate levels, those who had hyperlactatemia seemed to have higher odds of delirium after 24 h of ICU admission. In addition, 69.56% of persons’ serum lactate levels could return to normal levels within 24 h. The relationship between T1 lactate and delirium suggests that it is necessary to continuously pay attention to lactate levels in clinical practice so as to timely adjust treatment or take appropriate measures to reduce the risk of delirium in ICU patients. Nevertheless, the causal associations of T0 and T1 lactate with delirium need to be further verified.

The underlying pathophysiology mechanisms that the association of serum lactate with delirium may be complex and diverse, where immunoinflammatory reaction, metabolic insufficiency, and cognitive disintegration are common hypotheses. Systemic inflammation results in diffuse microcirculatory impairment, including leukocyte adhering to vessel lining, endothelial cell swelling, perivascular edema, and narrowing of capillary diameters and lowers functional capillary density, leading to a decrease in nutritive perfusion and longer diffusion distances for oxygen (26). Acetylcholine synthesis is especially sensitive to low oxygen tension, and its deficiency plays an important role in delirium pathophysiology (26). Although patients with trauma may have severely reduced circulation and tissue hypoxia, their blood pressure may be in the normal range because of peripheral vasoconstriction (27). Similarly, in our study, delirium patients had significantly higher DBP and SBP than those without delirium, but hypertension was not significantly different between these two groups. In addition, the elevated serum lactate was considered a reflection of the elevation of intracerebral lactate concentration (28). As important cerebral metabolism substrates, intracerebral lactate and glucose will change similar concentrations when the brain’s energy consumption balance is disrupted (a state of hypo-glucose and hyper-lactate concentration), indicating an acute metabolism crisis (ACMC) (28). ACMC is recognized as the etiology of secondary brain injury, which can cause acute neurological function impairment, such as different degrees of impairment of attention, executive ability, cognitive ability, and emotional disorders (29). Our findings indicated that not only patients with trauma but also the general population in the ICU should be carefully continuously focused on the serum lactate level within and after 24 h of the ICU admission. Therefore, serum lactate as a rapidly and inexpensively measured parameter could have the potential to be an early indicator of delirium occurrence among ICU patients in clinical practice.

Lactate clearance rate has been proposed to be a more descriptive term for the global tissue states compared to single lactate concentration at one time point and could be optimal for predicting mortality in critical patients (30, 31). No study has reported the association of lactate clearance rate with delirium so far; however, its predictive value in mortality of other common diseases in the ICU has gained a lot of support (31, 32). The underlying mechanisms that lactate clearance rate had a negative association with both delirium and 30-day mortality in ICU patients may be multitudinous. Lactate clearance rate early in the hospital course may indicate the resolution of global tissue hypoxia and further reduce the mortality rate (33). In addition, early lactate clearance was significantly associated with a decreased level of pro-inflammatory biomarkers, suggesting that lactate clearance rate could be an indirect prognostic marker (34). Although there is no clear biological mechanism to support the association of lactate clearance rate with delirium, we speculated that a higher lactate clearance rate may reduce the risk of delirium by improving the relative pathways, such as inflammatory response and cerebrovascular changes. According to the study results, we hold the opinion that dynamic monitoring of lactate clearance rate (which is best to be maintained above 50%) in ICU patients may be valuable for reducing the risk of both delirium and 30-day mortality, especially in those who had baseline hyperlactatemia/lactic acidosis.

Subgroup analyses showed that among patients without hypertension, who did not use sedative drugs, or with SOFA score ≤ 3, T1 lactate level had a positive association with delirium odds. On the other hand, the association of higher lactate clearance rate with lower odds of delirium was found in age ≥ 65 years old, non-hypertension, ventilation, antibiotic drug, vasopressors, male, female, sepsis, sedative drug, and SOFA score subgroups. It has been reported that preexisting or non-modifiable risk factors for delirium include aging >65 years old, male sex, alcohol abuse, brain trauma, dementia, hypertension, polypharmacy, and multiple medical comorbidities (35–37). In the present study, maintaining a higher lactate clearance rate (more than 50%) in patients with advanced age, without hypertension, who received medication, or equipment-assisted treatment could reduce the odds of delirium after excluding those who had alcohol abuse, brain trauma, or dementia. In conclusion, our findings were consistent with those of previous studies mentioned above, which indicated that both serum lactate level at different time points and lactate clearance rate in general ICU patients should be focused, and the population with milder disease conditions or undergoing relatively comprehensive treatment should not be ignored.

There are some strengths and limitations of this research. The current study is the first to investigate the associations of serum lactate at different time points and lactate clearance rate with delirium and subsequent mortality risk in the early stage of the ICU stay. Meanwhile, on the basis of the MIMIC-IV database with a large sample size, we considered multi-dimensional influencing factors for analyses, and the results were relatively reliable. However, as a single-center retrospective study, it is hard to conclude causal associations of lactate and lactate clearance rate with delirium, and interpretation of the results is also limited by selection bias. In addition, due to the limitation of the MIMIC-IV database, we could not discuss more details on the median- and long-term prognoses of patients with delirium other than 30-day mortality.

## Conclusion

Higher lactate levels at different time points of the ICU admission were associated with higher odds of delirium, whereas keeping a higher lactate clearance rate may reduce the potential risk of delirium and subsequent short-term mortality in delirium patients. However, further prospective studies are still needed to clarify the causal associations of lactate and lactate clearance rate with delirium.

## Data availability statement

Publicly available datasets were analyzed in this study. The datasets generated and/or analyzed during the current study are available in the MIMIC IV database, https://mimic.physionet.org/iv/.

## Author contributions

XQ: Writing – original draft, Writing – review & editing. YS: Data curation, Writing – review & editing. YJ: Methodology, Supervision, Writing – review & editing. YX: Writing – review & editing.
