# Supplementary figures and images for: Associations of serum lactate and lactate clearance with delirium in the early stage of ICU: a retrospective cohort study of the MIMIC-IV database
Source: Front Neurol. 2024 Jul 1;15:1371827. doi: 10.3389/fneur.2024.1371827 (PMC11246852; doi:10.3389/fneur.2024.1371827)

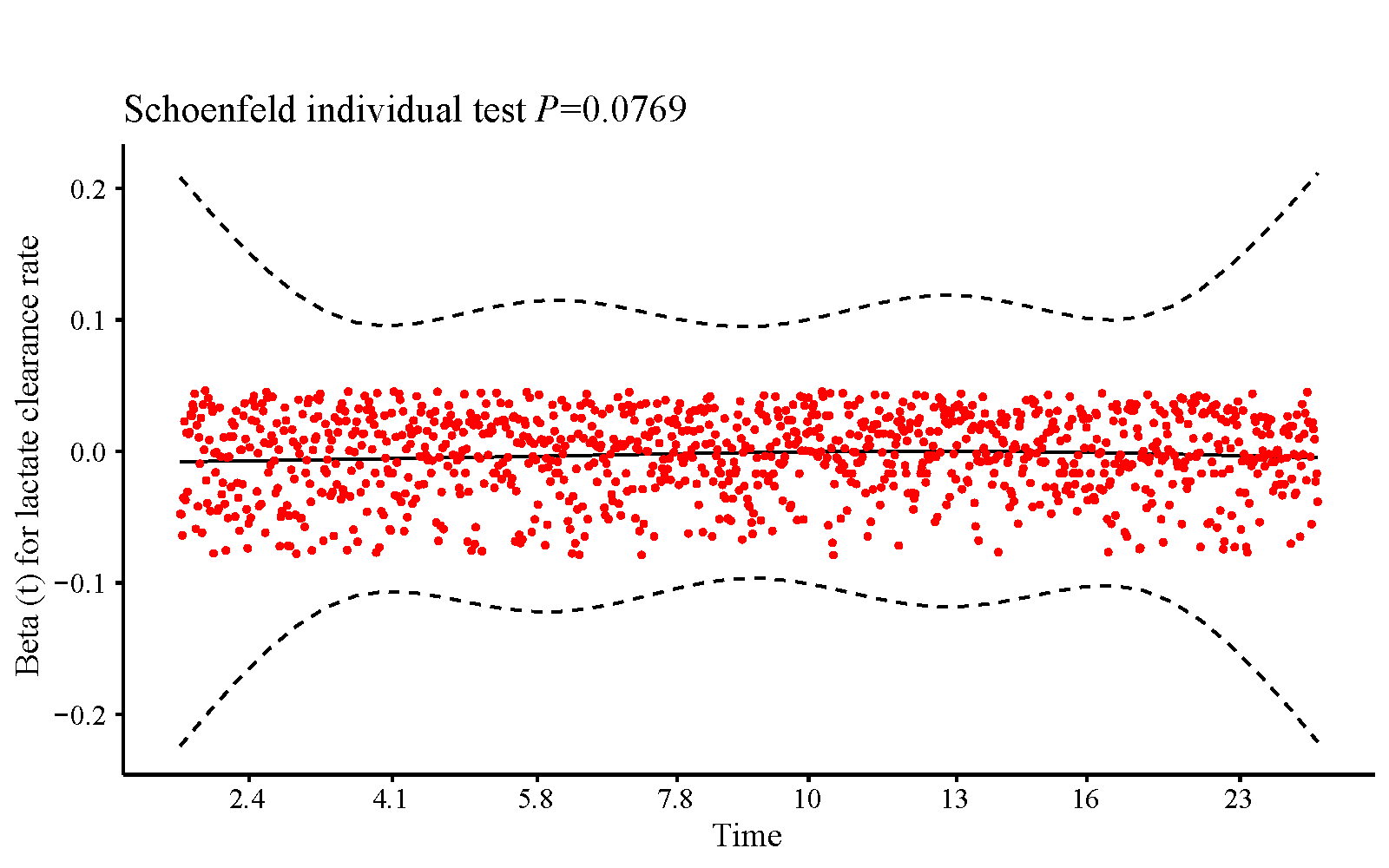

Supplement: Supplementary file 1 [file Image_1.TIFF]

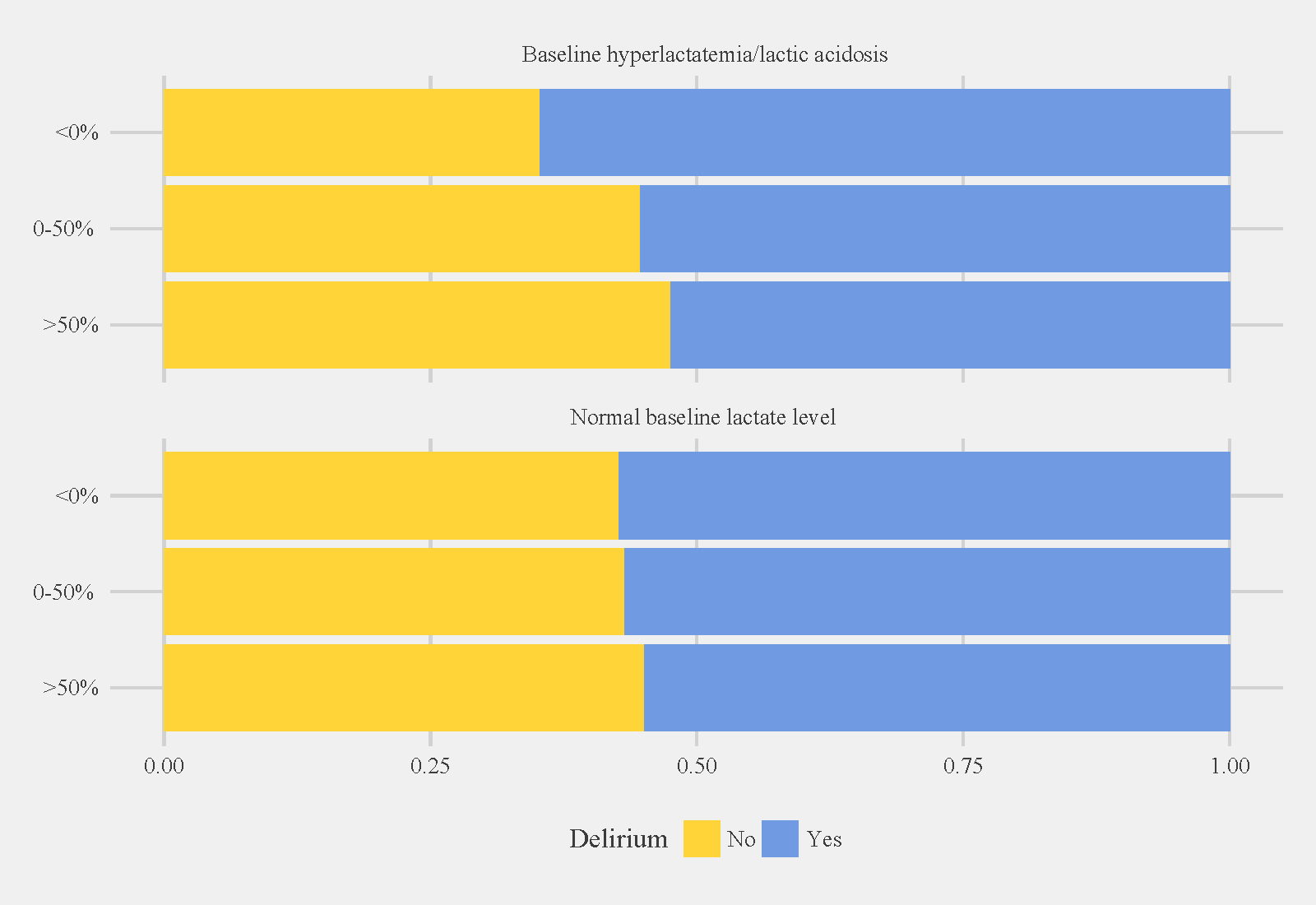

Supplement: Supplementary file 2 [file Image_2.TIFF]
