# Supplementary material for: Associations of serum lactate and lactate clearance with delirium in the early stage of ICU: a retrospective cohort study of the MIMIC-IV database
Source: Front Neurol. 2024 Jul 1;15:1371827. doi: 10.3389/fneur.2024.1371827 (PMC11246852; doi:10.3389/fneur.2024.1371827)
Supplement: Supplementary file 3 [file Table_1.DOCX]

**Table S1. Assessment of the multicollinearity among study variables in logistic regression models**

| Variables | VIF |
| --- | --- |
| Lactate clearance rate | 1.023531 |
| Race | 1.016776 |
| HR | 1.173037 |
| DBP | 1.307788 |
| SBP | 1.290495 |
| RR | 1.114791 |
| Temperature | 1.079702 |
| SPO_2_ | 1.065512 |
| pH | 1.149735 |
| RDW | 1.118812 |
| Platelet | 1.076318 |
| Cr | 1.436618 |
| INR | 3.019621 |
| PT | 3.025993 |
| Bun | 1.476295 |
| Bicarbonate | 1.168206 |
| Na | 1.039345 |
| Glucose | 1.063001 |
| Sofa | 1.259807 |
| CCI | 1.230117 |
| GCS | 1.036167 |
| Sepsis | 1.044638 |
| CKD | 1.173786 |
| Liver disease | 1.125856 |
| Ventilation | 1.136939 |
| Vasopressors | 1.129693 |
| VAP | 1.006956 |
| Sedative drug | 1.252033 |
| Antibiotic drug | 1.053992 |

HR: heart rate, DBP: diastolic blood pressure, SBP: systolic blood pressure, RR: respiratory rate, RDW: red cell distribution width, Cr: creatinine, INR: international normalized ratio, PT: prothrombin time, Na: sodium, SOFA: the Sequential Organ Failure Assessment, CCI: the Charlson Comorbidity Index, GCS: the Glasgow Coma Scale, CKD: chronic kidney disease, VAP: ventilator-associated pneumonia.

**Table S2. Associations of lactate clearance rate with delirium in different lactate levels at T0**

| Variables | Model 1 | | Model 2 | | Model 3 | |
| --- | --- | --- | --- | --- | --- | --- |
|  | OR (95% CI) | P | OR (95% CI) | P | OR (95% CI) | P |
| **Normal baseline lactate level** | | | | | | |
| Lactate clearance rate | 0.963 (0.900-1.030) | 0.272 | 0.956 (0.893-1.023) | 0.194 | 0.962 (0.892-1.036) | 0.306 |
| Lactate clearance rate |  |  |  |  |  |  |
| <0% | Ref |  | Ref |  | Ref |  |
| 0-50% | 0.978 (0.855-1.120) | 0.749 | 0.985 (0.858-1.129) | 0.825 | 0.939 (0.805-1.095) | 0.421 |
| >50% | 0.918 (0.641-1.315) | 0.641 | 0.947 (0.659-1.362) | 0.770 | 0.901 (0.602-1.348) | 0.613 |
| **Hyperlactatemia/lactic acidosis** | | | | | | |
| Lactate clearance rate | 0.862 (0.802-0.926) | <0.001 | 0.886 (0.825-0.952) | <0.001 | 0.901 (0.835-0.973) | 0.008 |
| Lactate clearance rate |  |  |  |  |  |  |
| <0% | Ref |  | Ref |  | Ref |  |
| 0-50% | 0.705 (0.585-0.850) | <0.001 | 0.739 (0.611-0.893) | 0.002 | 0.792 (0.643-0.976) | 0.028 |
| >50% | 0.587 (0.486-0.709) | <0.001 | 0.634 (0.521-0.770) | <0.001 | 0.620 (0.500-0.768) | <0.001 |

OR: odds ratio, CI: confidence interval, Ref: reference.

Mode1 1: adjusted for race, HR, DBP, SBP, RR, SPO_2_, pH;

Model 2: adjusted for race, HR, DBP, SBP, RR, SPO_2_, pH, RDW, platelet, Cr, INR, PT, BUN, bicarbonate, Na;

Model 3: adjusted for race, HR, DBP, SBP, RR, SPO_2_, pH, RDW, platelet, Cr, INR, PT, BUN, bicarbonate, Na, SOFA, CCI, GCS, sepsis, CVD, liver disease, ventilation, vasopressors use, VAP, sedative drug use, and antibiotic drug use.
